# Community perceptions of pre-eclampsia in rural Karnataka State, India: a qualitative study

**DOI:** 10.1186/s12978-016-0137-9

**Published:** 2016-06-08

**Authors:** Marianne Vidler, Umesh Charantimath, Geetanjali Katageri, Umesh Ramadurg, Chandrashekhar Karadiguddi, Diane Sawchuck, Rahat Qureshi, Shafik Dharamsi, Peter von Dadelszen, Richard Derman, Shivaprasad Goudar, Ashalata Mallapur, Mrutyunjaya Bellad, for the Community Level Interventions for Pre-eclampsia (CLIP) India Feasibility Working Group

**Affiliations:** Department of Obstetrics and Gynaecology, and the Child and Family Research Unit, University of British Columbia, Vancouver, BC Canada; KLE University’s Jawaharlal Nehru Medical College, Belgaum, Karnataka India; Department of Obstetrics and Gynaecology, S Nijalingappa Medical College, Bagalkot, Karnataka India; Department of Community Medicine, S Nijalingappa Medical College, Bagalkot, Karnataka India; Division of Women and Child Health, Aga Khan University, Karachi, Sindh Pakistan; Department of Family Practice, Faculty of Medicine, University of British Columbia, Vancouver, BC Canada; Department of Obstetrics, Christiana Care, Wilmington, Delaware United States

**Keywords:** Perception, Culture, Seizures, Rural population, Pregnancy, Pre-eclampsia, Traditional, medicine, Hypertension, India, Attitude, Eclampsia

## Abstract

**Background:**

Maternal deaths have been attributed in large part to delays in recognition of illness, timely transport to facility, and timely treatment once there. As community perceptions of pregnancy and their complications are critical to averting maternal morbidity and mortality, this study sought to contribute to the literature and explore community-based understandings of pre-eclampsia and eclampsia.

**Methods:**

The study was conducted in rural Karnataka State, India, in 2012–2013. Fourteen focus groups were held with the following community stakeholders: three with community leaders (*n* = 27), two with male decision-makers (*n* = 19), three with female decision-makers (*n* = 41), and six with reproductive age women (*n* = 132). Focus groups were facilitated by local researchers with clinical and research expertise. Discussions were audio-recorded, transcribed verbatim and translated to English for thematic analysis using NVivo 10.

**Results:**

Terminology exists in the local language (Kannada) to describe convulsions and hypertension, but there were no terms that are specific to pregnancy. Community participants perceived stress, tension and poor diet to be precipitants of hypertension in pregnancy. Seizures in pregnancy were thought to be brought on by anaemia, poor medical adherence, lack of tetanus toxoid immunization, and exposure in pregnancy to fire or water. Sweating, fatigue, dizziness-unsteadiness, swelling, and irritability were perceived to be signs of hypertension, which was recognized to have the potential to lead to eclampsia or death. Home remedies, such as providing the smell of onion, placing an iron object in the hands, or squeezing the fingers and toes, were all used regularly to treat seizures prior to accessing facility-based care although transport is not delayed.

**Conclusions:**

It is evident that ‘pre-eclampsia’ and ‘eclampsia’ are not well-known; instead hypertension and seizures are perceived as conditions that may occur during or outside pregnancy. Improving community knowledge about, and modifying attitudes towards, hypertension in pregnancy and its complications (including eclampsia) has the potential to address community-based delays in disease recognition and delays in treatment that contribute to maternal and perinatal morbidity and mortality. Advocacy and educational initiatives should be designed to target knowledge gaps and potentially harmful practices, and respond to cultural understandings of disease.

**Trial registration:**

NCT01911494

**Electronic supplementary material:**

The online version of this article (doi:10.1186/s12978-016-0137-9) contains supplementary material, which is available to authorized users.

## Background

Reduction of maternal and perinatal mortality is a global priority, particularly in low-and-middle-income countries (LMICs) where more than 99 % of these deaths occur. Increasingly, it is recognized that for every woman who dies, up to 25 others suffer a potential life-changing morbidity [1].

India accounts for 17 % of all maternal deaths worldwide [2]. Since 2004, efforts to lower maternal mortality, such as the National Rural Health Mission (NRHM) [3], have had some success. The latest estimated maternal mortality ratio (MMR) of India is 190- 282/100,000 live births but there is regional variation; the MMR in Karnataka state is below the national average at 144/100,000 live births [4, 5], but it is still the highest in South India. Also, the current MMR has not reached the global Millennium Development Goal 5 of a ¾ reduction in maternal mortality, equivalent to 109/100,000 live births in India [6].

The hypertensive disorders of pregnancy (HDPs) are responsible for ~14 % of all maternal deaths, with little change in rates in recent years [7]. The most dangerous of the HDPs, pre-eclampsia, is typically defined as the onset of new hypertension with significant proteinuria in pregnancy [8].

While management of pre-eclampsia involves the use of antihypertensive therapy to control blood pressure and steroids to accelerate fetal pulmonary maturity at gestational ages before 34 weeks, the only effective cure is delivery of the placenta [9]. Even then, the disease can worsen, or appear for the first time, postpartum. Although the disease can affect almost any organ in the body, involvement of the brain with seizures, known as eclampsia, poses particular risk for mother and baby.

Maternal deaths and complications in pre-eclampsia (and in general) have been attributed in large part to delays in recognition of illness and in timely transport to facilities for treatment [10]. As such, community perceptions of pre-eclampsia and eclampsia are critical to averting maternal morbidity and mortality. The limited literature suggests widespread misconceptions globally regarding presentation, cause, and appropriate treatment of pre-eclampsia [11]. Most research has focused on women’s perceptions without exploring those of other key decision-makers. For example, in Karnataka State, India, only 13 % of pregnant women had accurate knowledge about pre-eclampsia [12]. Also, in a systematic review of maternal health interventions in resource limited countries, only 37 % of programmes included community-based education and communication [13].

As part of the feasibility work for the Community Level Interventions for Pre-eclampsia (CLIP) (NCT01911494) cluster randomised controlled trial [14], community perceptions of pre-eclampsia and eclampsia in Karnataka State, India were explored. This study aimed to inform implementation of the CLIP intervention, as well as to inform researchers, program managers, health care providers and policy makers about how best to address delays in triage, transport and treatment of women with pre-eclampsia and eclampsia.

## Methods

### Study area

**Fig. 1:**
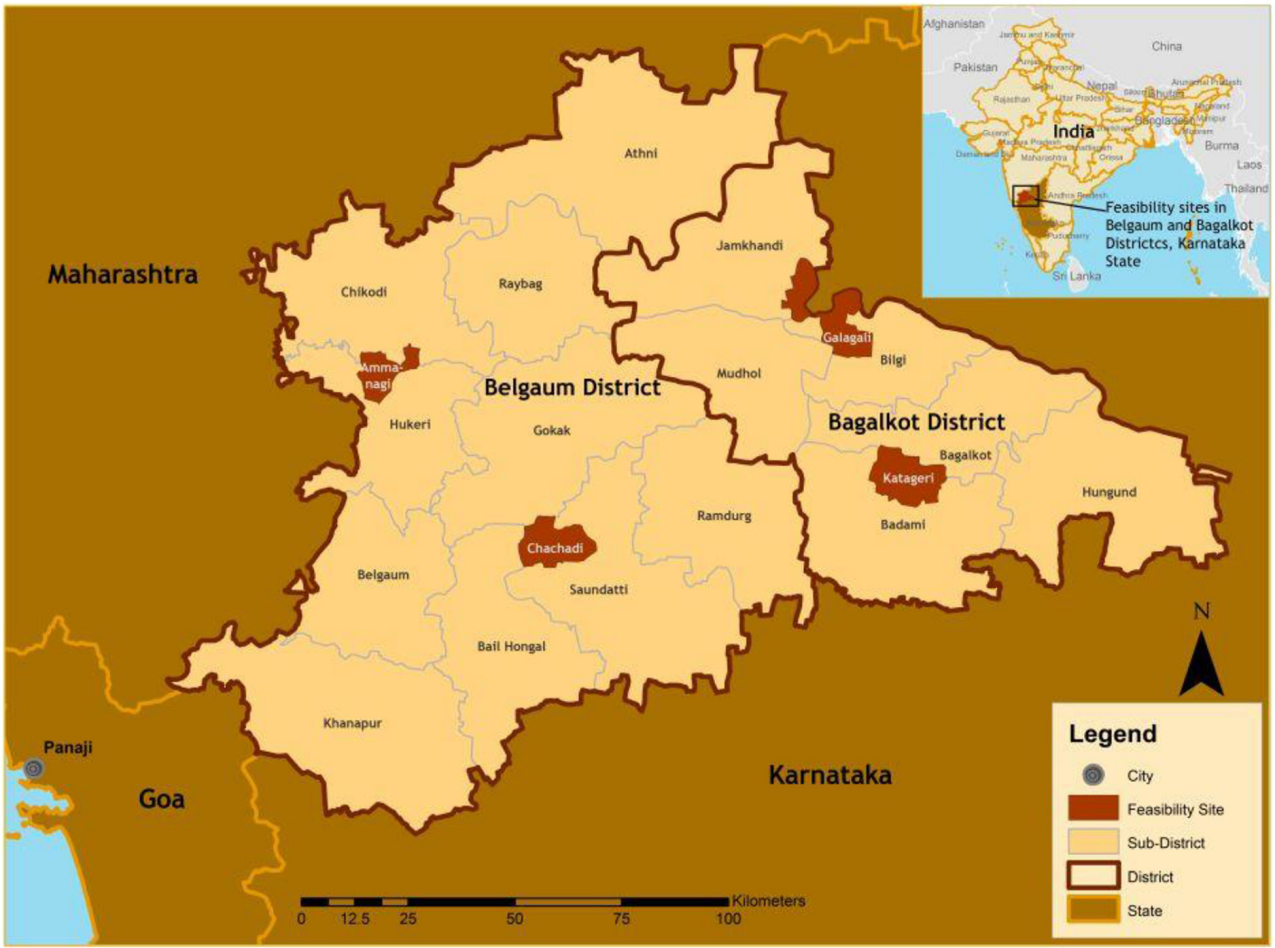
Map of study sites, Karnataka State, India

This study was conducted in Belgaum and Bagalkot Districts of Karnataka, in South India (Fig. 1). Social and health indicators of Karnataka are generally lagging compared with other southern Indian States. This gap is explained by fragmentation of the social, political and administrative structure, prevailing mismanagement, and a lack of state commitment and leadership regarding maternal health [3, 15]. Karnataka shares similar cultural practices with other Indian states, including a strong culture of patriarchy, limited decision-making power for women, early age of marriage, and high fertility rates. The health care infrastructure is often inadequate, particularly in rural areas where access to timely health services is a challenge. For additional study site characteristics see Table 1.

**Table 1:** Study site characteristics

India Characteristics	
Population	1,210,193,444
Size (Km^2^)	3,287,590
Number of states	28
Number of union territories	7
Predominant language	Hindi
Predominant religion	Hindu
Literacy rate of women 15–49 years	55 %
Karnataka State Characteristics	
Population	61,130,704
Size (Km^2^)	191,791
Number of districts	30
Predominant language	Kannada
Predominant religion	Hindu
Literacy rate of women 15–49 years	60 %
Site Characteristics	
Cumulative population	86,470
Cumulative size (Km^2^)	608
Number of clusters sampled	4
Number of villages	34

(International Institute for Population Sciences and Macro International, 2007)

### Data collection

Data were collected as part of a larger study aimed at assessing the feasibility of implementing a community-based treatment package for pre-eclampsia by community health care workers (NCT01911494) [14]. A detailed description of the methods has been published [16].

In brief, focus group discussions were conducted to elucidate community views from: community leaders, male and female decision-makers, and women of reproductive age. These groups were chosen as influential members of the community and family, particularly regarding health knowledge and decision-making. Mothers, mothers-in-law and husbands are typically responsible for household decisions and are influential in the beliefs and practices adopted by other household members. Male and female decision-makers were approached for participation when they accompanied women of reproductive age to the health centre. Focus group discussions were conducted with each stakeholder group separately at primary health centres, between January and March 2013. Discussions were in the local language, Kannada, to best promote interaction between community members. A semi-structured guide was used for all discussions, this approach allowed interviewers to tailor questions and probes as needed for differing groups; open-ended questions promoted free and open discussion among participants. The discussion guide was developed to meet the objectives of this study and had been created to fit the cultural context of South India. Focus group leaders had backgrounds in public health, community medicine or obstetrics. These professional and educational backgrounds made facilitators well-equipped for discussions on this topic, all also underwent study-specific training regarding qualitative data collection. Further meetings were to be scheduled thereafter if data saturation was not reached.

**Fig. 2:**
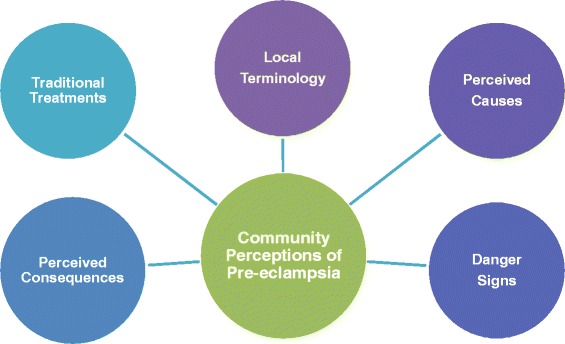
Thematic analysis categories

All discussion notes and audio recordings were transcribed verbatim and translated to English for analysis using NVivo 10. Coding was done by one researcher (MV), after which all coded transcripts and thematic associations were cross-checked by the local research team to resolve any discrepancies and reach consensus. Using deductive reasoning, results were grouped into predetermined categories of key themes related to community perceptions of pre-eclampsia (Fig. 2). Inductive reasoning was used to incorporate new and unexpected ideas. This produced a comprehensive analysis structure to reflect the richness and variety of responses. *A priori*, the following thematic categories were to be explored for pre-eclampsia: local terminology, perceived cause, warning (‘danger’) signs, perceived consequences, and traditional treatments.

This study was approved by ethics review committees at the University of British Columbia, Vancouver Canada (H12-00132) and KLE University, Belgaum India (MDC/IECHSR/2011-12).

## Results

There were 14 focus group discussions with 219 individuals: community leaders (3 groups, 27 participants), male decision-makers (2 groups, 19 individuals), female decision-makers (3 groups, 41 individuals), and women of reproductive age (6 groups, 132 individuals). The age of participants ranged from 18 to 65 years, and occupation was generally “agricultural or domestic labour”. There were diverse educational backgrounds, some with no formal schooling, particularly in groups of female decision-makers and women of reproductive age, and several with University completion. Most women of reproductive age who participated were pregnant at the time (79 %), and over half had at least one child under the age of 5 (66 %) (Table 2).

**Table 2:** Focus group characteristics

Number	N participants	Age (yr) Median [range]	Occupation 1 Housewife 2 Labourer 3 Employee 4 Self-employed 5 Other	Child <5 years	Pregnant	Education 1 No formal schooling, cannot read or write 2 No formal schooling, can read and write 3 Primary school incomplete 4 Primary school complete 5 Secondary school incomplete 6 Secondary school complete 7 Pre-university incomplete 8 Pre-university complete 9 University incomplete 10 University complete 11 Postgraduate 12 Don’t know	Relationship to woman 1 Husband 2 Father 3 Father-in-law 4 Mother-in-law 5 Mother 6 Other
Community Leaders							
1	7	36 [31,48]	1 = (*N* = 1) 2 = (*N* = 6)	*Not asked*	*Not asked*	1 = (*N* = 1) 6 = (*N* = 1) 8 = (*N* = 2) 10 = (*N* = 3)	*Not asked*
2	10	36 [24,51]	1 = (*N* = 3) 4 = (*N* = 3) 5 = (*N* = 4)	*Not asked*	*Not asked*	1 = (*N* = 1) 3 = (*N* = 1) 4 = (*N* = 1) 5 = (*N* = 1) 6 = (*N* = 3) 8 = (*N* = 1) 9 = (*N* = 1) 10 = (*N* = 1)	*Not asked*
3	10	*Not known*	*Not known*	*Not asked*	*Not asked*	*Not known*	*Not asked*
Male Decision-Makers							
1	8	26 [18,57]	2 = (*N* = 4) 3 = (*N* = 1) 4 = (*N* = 1) 5 = (*N* = 2)	*Not Applicable*	*Not Applicable*	1 = (*N* = 4) 3 = (*N* = 2) 6 = (*N* = 2)	1 = (*N* = 4) 3 = (*N* = 2) 6 = (*N* = 2)
2	11	49 [33,59]	3 = (*N* = 2) 5 = (*N* = 9)	*Not Applicable*	*Not Applicable*	3 = (*N* = 4) 5 = (*N* = 1) 6 = (*N* = 1) 10 = (*N* = 1) 12 = (*N* = 4)	2 = (*N* = 4) 3 = (*N* = 3) 6 = (*N* = 4)
Female Decision-Makers							
1	10	45 [30,60]	1 = (*N* = 9) 2 = (*N* = 1)	*Not Applicable*	*Not Applicable*	1 = (*N* = 8) 4 = (*N* = 2)	4 = (*N* = 6) 5 = (*N* = 2) 6 = (*N* = 2)
2	18	45 [28,65]	1 = (*N* = 5) 3 = (*N* = 1) 4 = (*N* = 1) 5 = (*N* = 11)	*Not Applicable*	*Not Applicable*	1 = (*N* = 3) 2 = (*N* = 1) 3 = (*N* = 1) 12 = (*N* = 13)	*Not Known*
3	13	48 [30,65]	1 = (*N* = 13)	*Not Applicable*	*Not Applicable*	3 = (*N* = 1) 5 = (*N* = 1) 12 = (*N* = 11)	4 = (*N* = 7) 5 = (*N* = 1) 6 (*N* = 5)
Women of Reproductive Age							
1	55	*Not Known*	*Not Known*	*Not Known*	*Not Known*	*Not Known*	*Not Known*
2	16	25 [20,30]	1 = (*N* = 16)	56 %	75 %	3 = (*N* = 3) 4 = (*N* = 1) 5 = (*N* = 1) 6 = (*N* = 2) 8 = (*N* = 2) 10 = (*N* = 1) 12 = (*N* = 6)	*Not Applicable*
3	14	23 [18,30]	1 = (*N* = 9) 2 = (*N* = 5)	36 %	86 %	1 = (*N* = 3) 3 = (*N* = 3) 4 = (*N* = 1) 5 = (*N* = 1) 6 = (*N* = 3) 8 = (*N* = 2) 10 = (*N* = 1)	*Not Applicable*
4	17	*Not Known*	1 = (*N* = 17)	88 %	100 %	*Not Known*	*Not Applicable*
5	14	22 [18,58]	1 = (*N* = 12) 3 = (*N* = 2)	71 %	50 %	3 = (*N* = 3) 5 = (*N* = 3) 6 = (*N* = 3) 10 = (*N* = 2) 12 = (*N* = 3)	*Not Applicable*
6	16	20 [19, 26]	1 = (*N* = 16)	69 %	63 %	3 = (*N* = 7) 4 = (*N* = 4) 5 = (*N* = 1) 6 = (*N* = 3) 8 = (*N* = 1)	*Not Applicable*

### Local terminology

Hypertension in pregnancy was referred to as ‘*BP*’ (blood pressure) in all stakeholder groups. In contrast, there were several terms used to describe convulsions in pregnancy or eclampsia (Table 3). Most commonly eclampsia was described as ‘*jhataka’* or ‘*fits’*. Other terms used were ‘*lakawa*’ and ‘*ardhangi*’ (meaning paralysis) or ‘*nanju agide*’ (meaning infection).

**Table 3:** Local terms for ‘eclampsia’

Local terms for ‘eclampsia’		
Jhataka Zataka	Moorcha roga Mala roga	Lakawa
Pepri	Nanju agide	Ghali shaka
Fits	Shatibyani Sete bene	Ardhangi

The community members had little understanding of pre-eclampsia or eclampsia as pregnancy complications. Eclampsia was often not differentiated from general convulsive disorders. Some respondents believed pre-eclampsia and eclampsia were related while others were unsure or did not believe this. However, even those who recognized this relationship often did not know the mechanism or direction of the association.

*“If blood pressure is high only then fits will occur” [community leader]*

### Perceived causes

The vast majority of respondents stated that hypertension in pregnancy was due to diet and tension or stress, particularly related to marital conflict or concerns that the fetus may be female. Poor diet was described as those high in salt or sugar and with excess fried and oily foods. Some felt hypertension in pregnancy had medical origins, while others believed the causes to be socio-economic and cultural (Table 4). By and large respondents expressed similar understandings of the causes of pre-eclampsia, however, it should be noted that male-decision makers provided the fewest responses, likely related to their lack of familiarity with the condition.

**Table 4:** Perceived causes of ‘pre-eclampsia’ and ‘eclampsia’

Causes of ‘pre-eclampsia’	Malnutrition	Martial problems	Stress	Tension
	Changes in body system	Medication side-effect	Poor adherence to medication	Lack of regular check-ups
	Lack of physical activity	Short temper	Poverty	Poor diet
	Home remedies	Reduced blood	Lack of water	Worries related to sex of baby
	Family problems	Anaemia		
Causes of ‘eclampsia’	Anaemia	Lack of energy	Hereditary	Blood pressure
	Poor adherence to medication	Malnutrition	Exposure to fire, water or heat	Lack of regular check-ups
	Lack of Tetanus Toxoid vaccination	Tension	Child marriage	Exposure to cold
	Fear

*“Those who worry more about their family they will have more BP” [female decision-maker]*

*“If they have disturbed food habits…like eating less or not eating properly or eating only limited food then they may have weakness and that may cause BP” [woman of reproductive age]*

Some gave similar responses for eclampsia; however, anaemia, poor medical adherence, not being immunized against tetanus, and exposure to fire or water in pregnancy were also commonly mentioned. It was described that a lack of regular antenatal attendance and adherence to medical advice and treatment could result in seizures in pregnancy (Table 4).

*“If they go near water …then they will get fits” [woman of reproductive age]*

### Danger signs

The most commonly reported signs or symptoms of pre-eclampsia were sweating, fatigue, dizziness-unsteadiness, swelling, anger, and talking too much. Respondents had a more challenging time explaining the danger signs of eclampsia, and often stated signs associated with active convulsions: frothing, shaking, eyes rolling upwards, clenching teeth, and tongue biting.

*“I witnessed one woman having fits, I saw that woman in hospital having labour pains and suddenly she had fits. Her hand, leg and body started shaking, became stiff, she bit her tongue, her eyeballs rolled up” [male decision-maker]*

### Perceived consequences

The most serious and most commonly cited consequences of hypertension or seizures in pregnancy were death of the mother and/or infant. Preterm delivery was also often mentioned. Concerns were raised about the need for Caesarean deliveries by women of reproductive age, which was felt to be highly undesirable. Seizures and heart-related problems were thought to be consequences of pre-eclampsia. Many participants from all groups professed ignorance about the consequences of eclampsia (Table 5).

**Table 5:** Outcomes for pre-eclampsia and eclampsia

	Outcomes affecting the mother	Outcomes affecting the infant
Pre-eclampsia	Mental imbalance	Stress
	Heart complications	Meconium aspiration
	Delivery complications	Delayed delivery
	Delayed delivery	Death
	Miscarriage	Stunted growth
	Bleeding	
	Death	
	Damage to the uterus	
	Weakness	
	Anaemia	
	Seizures	
	Coma	
	Falls	
	Fatigue	
	Paralysis	
	Brain tumours	
	Swelling	
Eclampsia	Death	Death
	Miscarriage	Injury
	Injury

*“High BP can endanger the life of a woman; it may lead to death of a woman which in turn is a problem for us” [female decision-maker]*

There were differences of opinions regarding the severity of pre-eclampsia and eclampsia, nevertheless many felt these conditions could be fatal.

### Traditional treatments

Neither traditional nor home-based remedies were reported for treating hypertension in pregnancy, although a few participants recommended restricting dietary salt. Various home remedies were reported in all focus group discussions for seizures in pregnancy, either during or immediately following a seizure, including providing the ‘smell of an onion’, placing an iron object or keys in the hand, and squeezing or rubbing the fingers and toes. Many also recommended that an object should be placed between a woman’s teeth to avoid biting her tongue. It was noted that the described practices typically do not prevent or delay transfer to hospital.

*“For the time being, we should give any iron object in her hand and then we should take her to hospital” [woman of reproductive age]*

## Discussion

There was very limited understanding of pre-eclampsia and eclampsia in these communities. In the two Districts of rural Karnataka, eclampsia was not differentiated from other convulsive disorders. Most did not realize pre-eclampsia and eclampsia are related, and those who did recognize this relationship often did not know its aetiology or that eclampsia progresses from pre-eclampsia. Local terms exist for hypertension and convulsions, some of which accurately, and others of which inaccurately, describe these conditions. Significant knowledge gaps were evident, particularly regarding warning signs, causes, and outcomes. No traditional remedies were reported for pre-eclampsia; however, there are a number of home-based remedies given to women who experience seizures in pregnancy.

The finding, that women had limited knowledge of pre-eclampsia and the associated signs and symptoms, is consistent with results from Nigeria and Pakistan [16–18]. Other studies, however, have found greater awareness of the condition [11, 19–21]. Attitudes expressed by participants regarding the cause of pre-eclampsia reflect the cultural dynamics with prevalent worry of giving birth to a female child. Pre-eclampsia was linked to exposure to fire or water, a myth that prevailed among many of the participants in the study. Similar to our study, in Nigeria and Brazil, pre-eclampsia was attributed to mental stress or diet, though they also attributed the condition to heredity [11, 22]. El-Nafty & Omotara [19] found four common explanations for causes of eclampsia in Nigeria: supernatural causes, malnutrition, heredity, and early age of marriage. In Bangladesh, community members thought eclampsia was due to exposure to a cold environment, physical weakness from malnutrition, hypertension, lack of tetanus immunization, and supernatural causes [20]. Our study findings are also consistent with other study results wherein communities perceive hypertensive disorders of pregnancy to be serious and potentially harmful conditions [20–23]. This high level or perceived severity is likely to influence health care seeking as described by the health belief model and these findings [24].

This study was conducted with a high degree of rigour in close collaboration with local and international experts and with guidance from an international consortium of advisors. These findings represent a comprehensive view of the community’s beliefs and practices due to participation with various stakeholders. The study procedures, including training of facilitators, careful translation of guides, and expert guidance all contribute to the strengths of the study.

As many participants were identified through primary health centre networks, women who did not interact with the health system may have been less likely to be included in the sample. The non-probabilistic sampling of this study inhibits the ability generalize findings, as indicated by the consistencies and inconsistencies of our findings with other studies as previously described. The results are further limited by the translation from Kannada to English for analysis, through which some of its meaning may have been lost. Although researchers were not providing direct clinical care to any of the participants, their dual role as researchers and clinicians may have introduced some bias and made participants more likely to respond favourably. Sensitive topics for discussion may have received less open discussion. While those conducting and facilitating the discussions were trained to garner equal input from all, focus group dynamics may have enhanced or hindered dialogue by some members.

Given that this study has identified knowledge gaps, there is a need to increase awareness of, and dispel common myths and misconceptions related to, the HDPs. Education and community engagement initiatives can assist women and communities to recognize the causes, risk factors, and warning signs associated with pre-eclampsia and eclampsia. Health professionals should facilitate dialogue with pregnant women and families in ways that will correct misinterpretations of the disease. To date, much of the education delivered through maternal and child health initiatives have targeted health facilities with limited evidence of the effectiveness [25]. Community-based education of diverse groups can reach a wider population. There is mounting evidence of the effectiveness of community and women’s groups to improve health outcomes [26, 27]. These types of approaches would strengthen the current efforts and mandate of the NRHM which aims at improving maternal and child health particularly for vulnerable populations in rural areas.

## Conclusions

Study results clearly indicate a need for further education of the community, moving from a narrow biomedical focus and incorporating the diverse cultural and social factors. Such an approach may positively influence knowledge and practice regarding pre-eclampsia and eclampsia. Greater community education on pre-eclampsia is necessary to achieve effective implementation of research and programmatic initiatives to improve morbidity and mortality.

## Additional file

Additional file 1: Peer review reports. (PDF 190 kb)

## Abbreviations

LMICs: low-and-middle-income countries
NRHM: National Rural Health Mission
MMR: maternal mortality ratio
HDPs: hypertensive disorders of pregnancy
CLIP: community level interventions for pre-eclampsia
