# Supplementary material for: Community perceptions of pre-eclampsia in rural Karnataka State, India: a qualitative study
Source: Reprod Health. 2016 Jun 8;13(Suppl 1):35. doi: 10.1186/s12978-016-0137-9 (PMC4943492; doi:10.1186/s12978-016-0137-9)
Supplement: Additional file 1: — Peer review reports. (PDF 190 kb) [file 12978_2016_137_MOESM1_ESM.pdf]

Referee's comments to the authors– this sheet WILL be seen by the author(s) and published with the article

Title	**Community perceptions of pre-eclampsia in rural Karnataka State, India: a qualitative study**
Author(s)	Marianne Vidler, Umesh Charantimath, Geetanjali Katageri, Umesh Ramadurg, Chandrashekhar Karadiguddi, Diane Sawchuck, Rahat Qureshi, Shafik Dharamsi, Peter von Dadelszen, Richard Derman, Shivaprasad Goudar, Ashalata Mallapur, Mrutyunjaya Bellad for the Community Level Interventions for Pre-eclampsia (CLIP) India Feasibility Working Group
Referee's name	Marianne Vidler

When assessing the work, please consider the following points, where applicable:

1. Is the question posed by the authors new and well defined?
2. Are the methods appropriate and well described, and are sufficient details provided to replicate the work?
3. Are the data sound and well controlled?
4. Does the manuscript adhere to the relevant standards for reporting and data deposition?
5. Are the discussion and conclusions well balanced and adequately supported by the data?
6. Do the title and abstract accurately convey what has been found?
7. Is the writing acceptable?

Please make your report as constructive and detailed as possible in your comments so that authors have the opportunity to overcome any serious deficiencies that you find and please also divide your comments into the following categories:

- Major Compulsory Revisions (which the author must respond to before a decision on publication can be reached)
- Minor Essential Revisions (such as missing labels on figures, or the wrong use of a term, which the author can be trusted to correct)
- Discretionary Revisions (which are recommendations for improvement but which the author can choose to ignore)

Where possible please supply references to substantiate your comments.

When referring to the manuscript please provide specific page and paragraph citations where appropriate.

General comments:

The authors provide an excellent background literature review and a strong and compelling need for the study.

Major compulsory revisions:
Methods: I would have appreciated more details on the methods. For example, why were these particular groups chosen, and what were the characteristics of this group? Among the female participants, how many had been pregnant and had pre-eclampsia? Who approached the male and female decision-makers at the health centre about participation? Who carried out the focus groups, and what were their qualifications? Did focus group leaders follow an interview guide/standard set of questions and were these consistent across the groups?

Please note that my copy did not contain the Tables or Figures.

Is it possible to put the ages of the respondents beside their corresponding quote? (This is customary in qualitative research).

The Results section is well-written, and chosen exerpts are very good for illustrating main points. However, I

(continue on the next sheet)

Continued:
believe that the authors could have discussed more whether there were differences vs similarities between the different groups regarding the major themes. For example, one might predict that male decision-makers may have different views given the highly paternalistic culture in which the study was carried out, but unfortunately, this was not mentioned in the manuscript.

Discussion: I would have liked to see the authors consider whether the participants beliefs about how to treat pre-eclampsia impact their delay to seek treatment or their willingness to accept conventional treatment.

Supplement Editor's comments
Please carefully revise the references and to follow the guidelines for references of the Journal. Also, in references not coming from Journals, please provide the link and the date that this link was accessed.
